# Targeting poor proteasomal function with radioiodine eliminates CT26 colon cancer stem cells resistant to bortezomib therapy

**DOI:** 10.1038/s41598-020-71366-3

**Published:** 2020-08-31

**Authors:** Jin Hee Lee, Kyung-Ho Jung, Jin Won Park, Seung Hwan Moon, Young Seok Cho, Kyung-Han Lee

**Affiliations:** 1 Department of Nuclear Medicine, Samsung Medical Center, Sungkyunkwan University School of Medicine, 50 Ilwon-dong, Gangnam-gu, Seoul, Korea; 2 Department of Health Sciences and Technology, SAIHST, Sungkyunkwan University, Seoul, Korea; 3 Scripps Korea Antibody Institute, Chuncheon-si, Gangwon-do Korea

**Keywords:** Cancer, Oncology

## Abstract

We tested the hypothesis that tumor response to conventional bortezomib (BTZ) treatment is enhanced by targeted radiotherapy of resistant cancer stem cells (CSCs) that have characteristically poor proteasome function. This was accomplished by augmenting ^131^I uptake through expression of a sodium-iodide symporter (NIS) fusion protein that accumulates in cells with low proteasome activity. The NIS gene fused with the C-terminal of ornithine decarboxylase degron (NIS-cODC) was cloned. Stably expressing CT26/NIS-cODC cells and tumorsphere-derived CSCs were evaluated for NIS expression and radioiodine uptake. CT26/NIS-cODC cells implanted into mice underwent PET imaging, and tumor-bearing mice were treated with BTZ alone or with BTZ plus ^131^I. CT26/NIS-cODC cells accumulated NIS protein, which led to high radioiodine uptake when proteasome activity was inhibited or after enrichment for stemness. The cell population that survived BTZ treatment was enriched with CSCs that were susceptible to ^131^I treatment, which suppressed stemness features. Positron emission tomography and uptake measurements confirmed high ^124^I and ^131^I uptake of CT26/NIS-cODC CSCs implanted in living mice. In CT26/NIS-cODC tumor-bearing mice, whereas BTZ treatment modestly retarded tumor growth and increased stemness markers, combining ^131^I therapy suppressed stemness features and achieved greater antitumor effects. The NIS-cODC system offer radioiodine-targeted elimination of CSCs that are tolerant to proteasome inhibition therapy.

## Introduction

A major challenge in cytotoxic cancer therapies is that not all tumor cells demonstrate equivalent sensitivity. Therefore, after cytotoxic drugs succeed in eliminating the bulk of the tumor, the limit of their effectiveness is met, and the surviving population propagates to drive cancer relapse^1^. Crucial contributors to this phenomenon are cancer cells with stemness properties that have preferential resistance to chemotherapeutics^2,3^.

Tumor relapse occurs through pre-existing resistant clones^4–6^, although oncogenic mutations induced by the cytotoxic agents might also contribute^7^. The recognition of a special tumor cell population that shares characteristics with normal stem cells is changing our perspective of cancer therapy^8^. These cancer stem cells (CSCs) are resistant to a variety of chemotherapeutics and have the capacity to self-renew and differentiate into all tumor cell types during relapse^2^. Therefore, improvement in cancer therapy crucially requires novel techniques that specifically target treatment-tolerant CSCs.

An emerging strategy in anticancer drug development is targeting essential tumor-supportive cellular machinery^9^, which includes regulations of division rate, transporter expression, and proteasomal activity. Differentiated tumor cells have high proteasome activity^10,11^ to help eliminate onco-suppressive proteins^12^, which is exploited by pharmacological proteasome inhibition for treatment^13,14^. Indeed, the proteasome inhibitor bortezomib (BTZ) has shown encouraging results in treating multiple myeloma and mantle cell lymphoma^15,16^. However, the clinical efficacy of proteasome inhibitors to treat solid tumors has been disappointing thus far^17^.

A previous study showed that proteasome inhibitors can destroy the bulk of a tumor, but they cannot eliminate drug-resistant CSCs that exhibit self-renewal^18^. In contrast to the bulk of tumor cells, CSCs, which have the capacity to propagate tumors and resist treatment, have poor proteasome function^19–25^. Downregulated proteasome activity could be a prerequisite for stabilizing the proteins required to maintain stem cell traits in these cells. In other words, BTZ can eliminate the bulk of tumor cells, which depend on high proteasome activity, but the surviving portion is likely to be composed of CSCs with poor proteasome function. New strategies to eliminate treatment-resistant cells are therefore needed to improve the efficacy of proteasome inhibitors, and a unique opportunity may be provided by eroding the advantages of perturbed proteasome function.

Our group recently demonstrated that the sodium iodine symporter (NIS) fused to the C-terminus of the ornithine decarboxylase (cODC) degron (NIS-cODC) instigates high levels of radioiodine uptake into cancer cells under proteasome suppression^26^. In this study, we hypothesized that the NIS-cODC system could be implemented to enable ^131^I-based therapeutic elimination of CSCs that contribute to resistance to BTZ treatment.

## Results

### CT26/NIS-cODC cells accumulate NIS and take up radioiodine under proteasome inhibition

**Figure 1:**
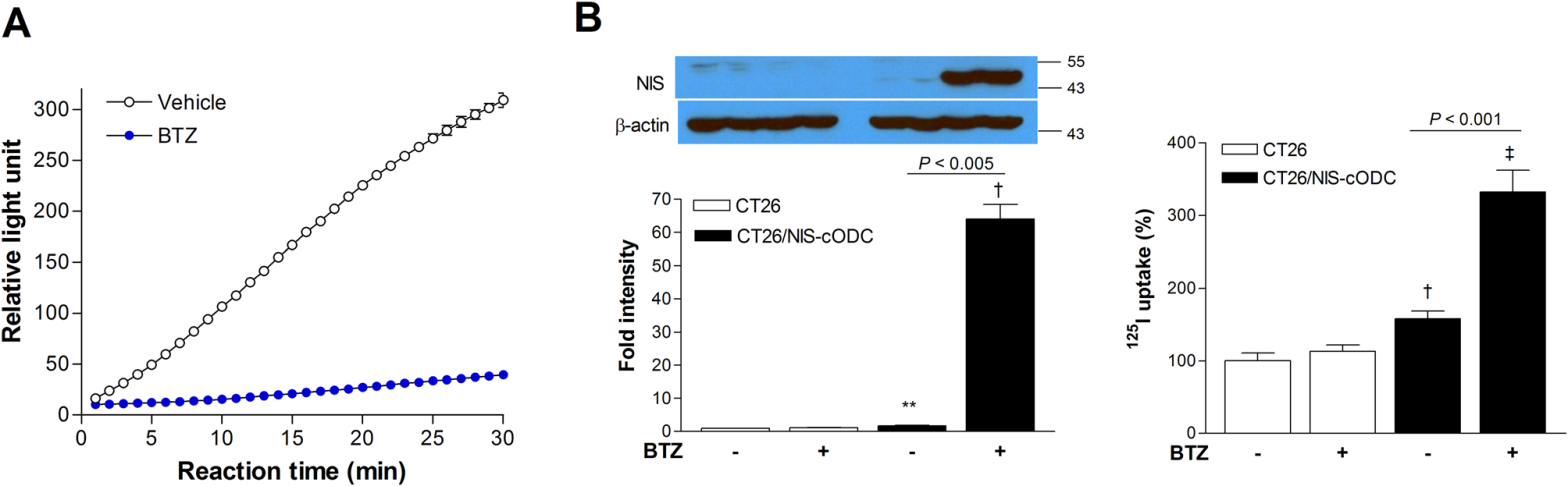
CT26/NIS-cODC cells increase NIS accumulation and radioiodine uptake under proteasome suppression. (**A**) Fluorometric assays of proteasome activity in cells under 36 h exposure to 100 nM BTZ. (**B**) Western blots and quantified band intensities of NIS expression normalized to β-actin bands (left) and ^125^I uptake (right) in CT26 and CT26/NIS-cODC under BTZ exposure (36 h at 100 nM, left; 16 h at 200 nM, right). Blots are cropped with single blot parts separated by space. For full length blot pictures, see supplement Fig. 1. Bars are mean ± SD of duplicate samples expressed as fold of controls (left) or mean ± SD of triplicate samples expressed as % of controls (right). **P < 0.01, ^†^*P* < 0.005, ^‡^*P* < 0.001 compared to control CT26 cells. BTZ: bortezomib; NIS: sodium-iodide symporter.

The proteasome activity of CT26 cells that constitutively overexpress NIS-cODC (CT26/NIS-cODC cells) was completely suppressed in the presence of 100 nM BTZ (Fig. 1A). In these cells, NIS protein was undetectable at baseline but markedly increased to 63.9 ± 4.4-fold of controls under BTZ exposure (*P* < 0.005; Fig. 1B). This contrasted with CT26 cells, wherein NIS expression was undetectable regardless of BTZ exposure (Fig. 1B). Similarly, ^125^I uptake was low in CT26/NIS-cODC cells at baseline but was substantially increased to 332.0 ± 29.8% of controls in the presence of BTZ (*P* < 0.001; Fig. 1B).

### CT26/NIS-cODC cells enriched for stemnesss accumulate NIS and have high radioiodine uptake

**Figure 2:**
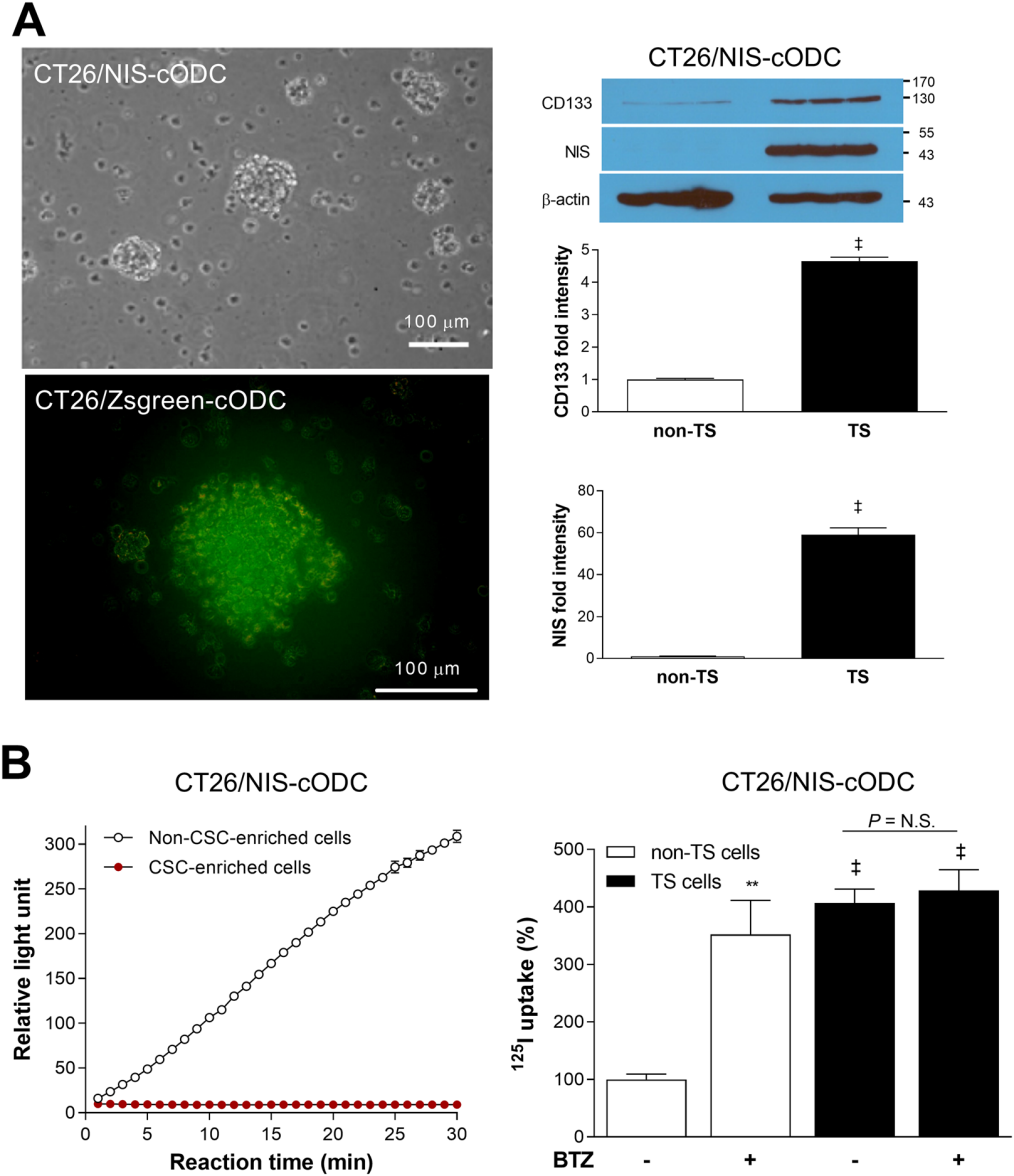
CT26/NIS-cODC CSCs have intrinsically low proteasome activity with high NIS accumulation and radioiodine uptake. (**A**) CT26 /NIS-cODC cells efficiently form tumorspheres (TS; left top); and TS of CT26/ZsGreen cells display increased fluorescence (left bottom). Western blots for CD133 and NIS in CT26/NIS-cODC cells grown as monolayers (non-TS) or as TS. Blots are cropped with single blot parts separated by space. For full length blot pictures, see supplement Fig. 2. Bars are the mean ± SD of band intensities normalized to β-actin from triplicate samples expressed as fold of non-TS controls. (**B**) Fluorometric assays of proteasome activity in CT26/NIS-cODC cells at baseline (left); and radioiodine uptake in cells at baseline or under BTZ exposure (16 h at 200 nM; right). Bars are the mean ± SD of uptake from triplicate samples expressed as % controls. **P < 0.01, ^‡^*P* < 0.001 compared to non-TS controls. CSC: cancer stem cell; BTZ: bortezomib; N.S.: not significant.

CT26/NIS-cODC cells efficiently formed tumorspheres after 10 days of culture on an ultra-low attachment plate in CSC selection medium (Fig. 2A). Tumorspheres of CT26 cells that constitutively express ZsGreen-cODC (CT26/ZsGreen-cODC cells) were highly fluorescent in the absence of BTZ, indicating inherently low proteasome activity (Fig. 2A). Tumorsphere-derived CT26/NIS-cODC cells displayed a complete abrogation of proteasome activity (Fig. 2B) and a 4.6 ± 0.1-fold increase of CD133 expression compared with monolayer-grown cells (*P* < 0.001; Fig. 2A), indicating enrichment for CSCs. These cells demonstrated a 59.0 ± 3.3-fold increase of NIS expression (*P* < 0.001; Fig. 2A) and high ^125^I uptake that reached 406.4 ± 24.5% of that of monolayer-grown control cells (*P* < 0.001; Fig. 2B). Furthermore, unlike monolayer-grown cells, the CSCs did not demonstrate a further increase of ^125^I uptake by BTZ exposure.

The lack of further increase of ^125^I uptake by BTZ treatment in tumorsphere-derived CT26/NIS-cODC cells is explained by their enrichment for CSCs, which have inherently low proteasome activity as confirmed in Fig. 2B. Low proteasome activity would suppress proteasomal degradation of cODC fused NIS protein, thereby negating any additional increase of NIS-cODC accumulation by the proteasome inhibitor BTZ. Hence, NIS-cODC accumulation and ^125^I uptake was high in CT26/NIS-cODC cells with little difference between baseline and after BTZ treatment.

### CT26/NIS-cODC cell populations that survive BTZ treatment are enriched with CSCs

**Figure 3:**
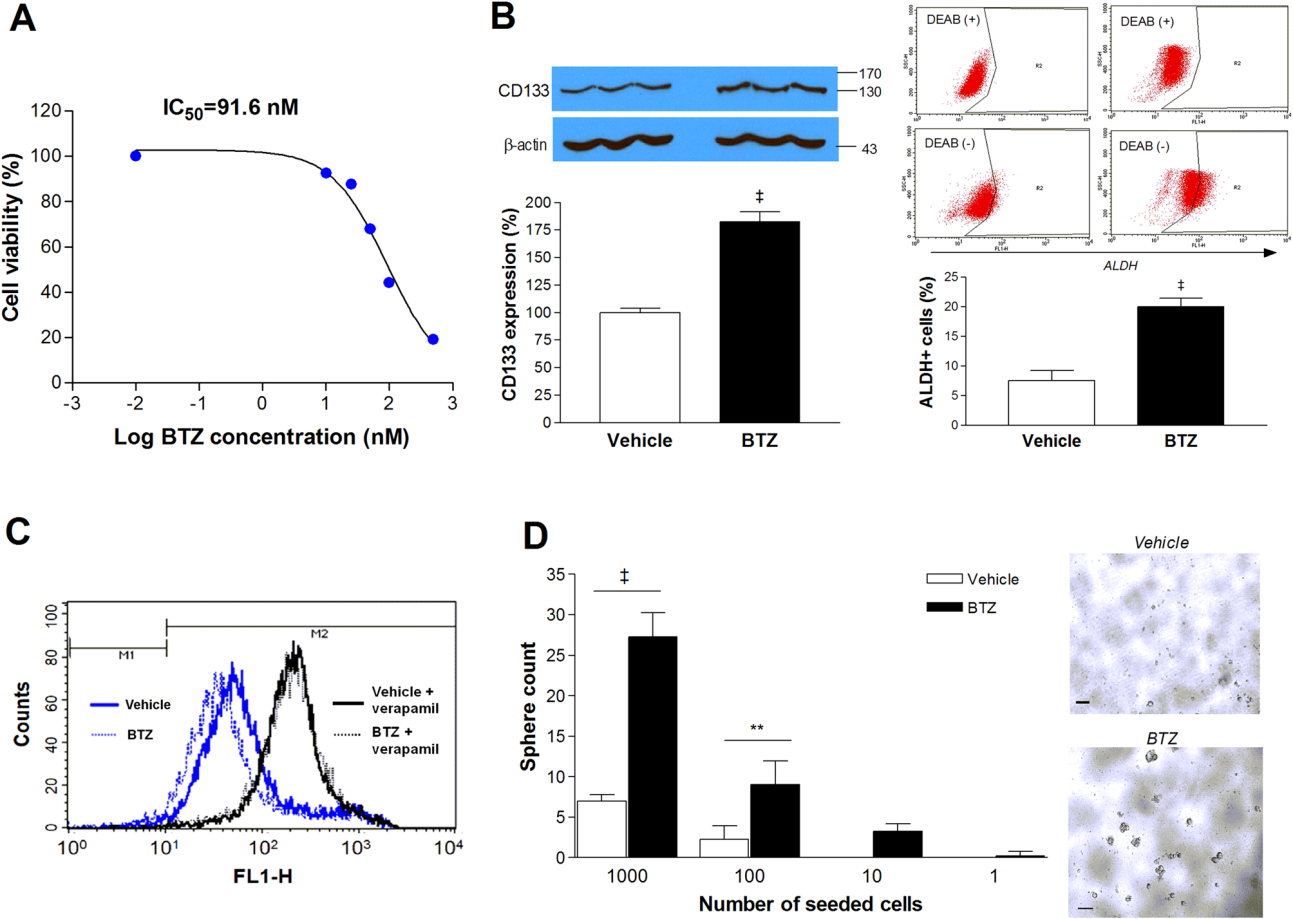
BTZ treatment of CT26/NIS-cODC cells result in a surviving fraction enriched for stemness. (**A**) The log concentration response curve for CT26/NIS-cODC cells survival after 84 h treatment with graded doses of BTZ. The half maximal inhibitory concentration (IC_50_) was determined from nonlinear regression curve fitting. Data are the mean ± SD of triplicate samples expressed as % control. (**B**) Western blots for CD133 (left) and FACS analysis of aldehyde dehydrogenase (ALDH) activity (right) in CT26/NIS-cODC cells that survived 7-day treatment with 50 nM BTZ. Blots are cropped with single blot parts separated by space. For full length blot pictures, see Supplement Fig. 3. Bars are mean ± SD obtained from triplicate samples of % band intensities normalized to β-actin bands (left) or proportion of ALDH-positive cells (right). (**C**) Efluxx-ID FACS analysis of p-glycoprotein-mediated dye efflux in CT26/NIS-cODC cells that survived BTZ treatment as above. (**D**) Limiting dilution assays for TS formation in CT26/NIS-cODC cells that survived BTZ treatment as above. Bars are the mean ± SD of the TS count of quadruplicate samples. **P < 0.01, ^‡^*P* < 0.001 compared to controls.

Survival of monolayer grown CT26/NIS-cODC cells was dose-dependently reduced by BTZ treatment, reaching 87.7 ± 0.7% and 67.9 ± 1.2% of the untreated level after 3 days of treatment with 25 nM and 50 nM BTZ, respectively. The half maximal inhibitory concentration (IC_50_) for BTZ was 91.6 nM (Fig. 3A). Cells that survived 7 days of treatment with 50 nM (freshly replaced every 2 days) were considered BTZ-resistant. When the cells tolerant to BTZ treatment were evaluated for stemness properties, western blots showed that CD133 expression was increased to 182.6 ± 8.8% of the baseline level (*P* < 0.001; Fig. 3B). In FACS assays, these cells showed a 2.7 ± 0.2-fold increase in Aldefluor positivity (*P* < 0.001) reaching 19.9 ± 1.4% from 7.5 ± 1.6% at baseline (Fig. 3B). In Efluxx-ID Green assays, a slight left shift with a small increase of stain-negative cells was observed (1.2 ± 0.2% vs. 2.5 ± 0.3%, *P* = 0.004; Fig. 3C), indicating slightly greater MDR1-mediated dye efflux. Furthermore, limiting dilution assays revealed that the number of tumorspheres formed from seeding 1,000 cells increased markedly, from 7.0 ± 0.8 to 27.2 ± 2.9 (*P* < 0.001; Fig. 3D). The minimum number of cells required for tumorsphere formation decreased from 100 cells for the untreated group to 10 cells for the BTZ-resistant group (Fig. 3D).

### ^131^I treatment augments the therapeutic efficacy of BTZ and suppresses cancer stemnesss

**Figure 4:**
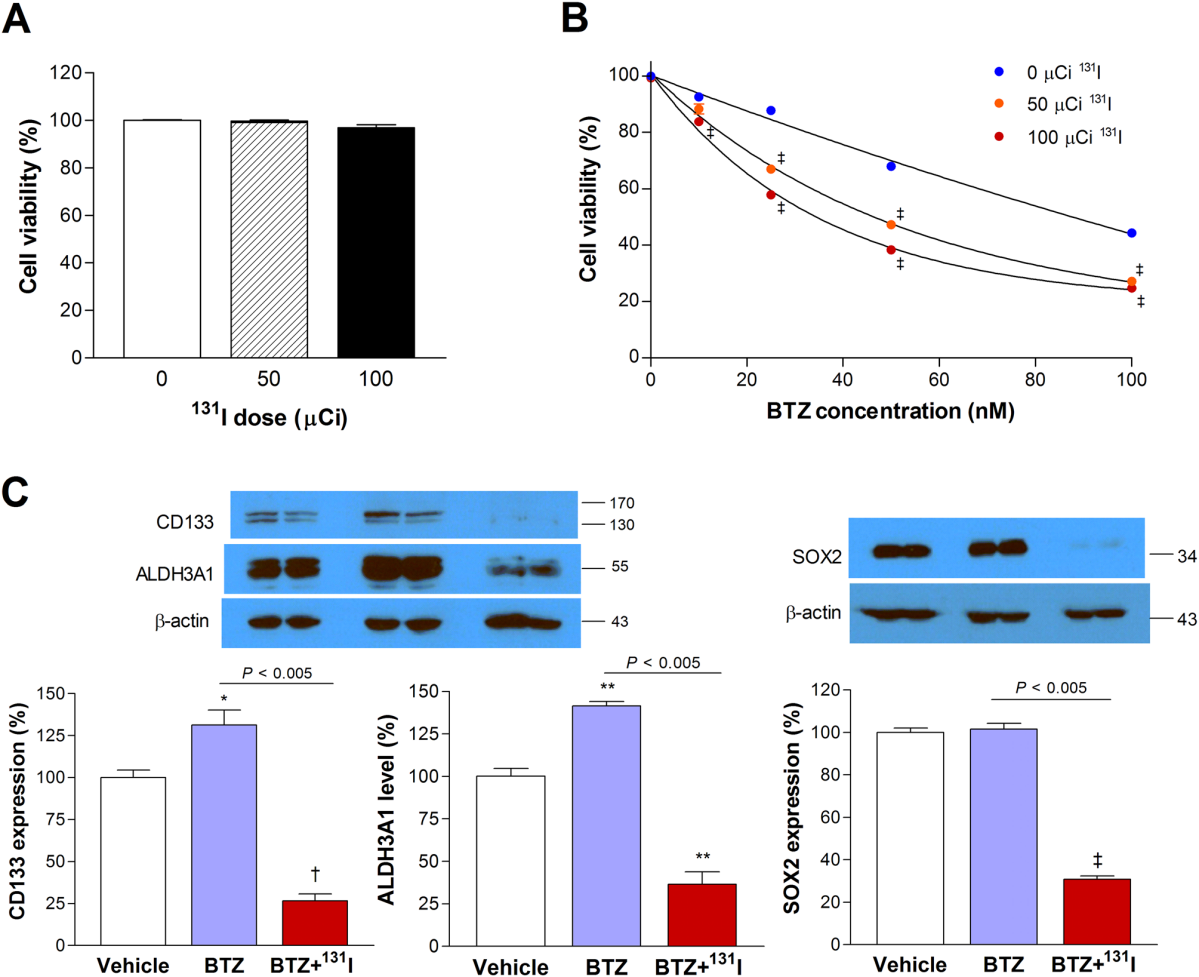
^131^I potentiates BTZ treatment efficacy and suppresses stemness properties in vitro. (**A**) Effects of ^131^I alone (48 h) on CT26/NIS-cODC cell viability. Bars are the mean ± SD of % survival from triplicate samples. (**B**) Survival of CT26/NIS-cODC cells after treatment with graded doses of BTZ with and without added ^131^I therapy. Data are the mean ± SD of the % survival from triplicate samples. ^‡^*P* < 0.001 compared to treatment with the same dose of BTZ alone. (**C**) Western blots for CD133, ALDH3A1, and SOX2 expression in CT26/NIS-cODC cells after 11 days of repeated treatment every 3 days with 25 nM BTZ alone or BTZ plus ^131^I (single injection at day 6). Blots are cropped with single blot parts separated by space. For full length blot pictures, see supplement Fig. 4. Bars are the mean ± SD of % band intensities normalized to β-actin bands obtained from duplicate samples. *P < 0.05, **P < 0.01, ^†^*P* < 0.005, ^‡^*P* < 0.001 compared to controls. ALDH3A1: Aldehyde dehydrogenase 3A1; SOX2: sex determining region Y-box 2.

We next investigated whether ^131^I therapy would enhance tumor treatment by eliminating BTZ-tolerant CSCs. We first showed that ^131^I alone did not influence CT26/NIS-cODC cell survival (Fig. 4A). This is because without tumorsphere formation or BTZ treatment, CT26/NIS-cODC cells have high proteasome activity that does not allow NIS-cODC accumulation. Since the culture medium was changed to fresh media at 9 h after I-131 was added, the level of radiation delivered to the cell interior would have been quite limited without cellular ^131^I uptake through NIS.

In contrast, adding ^131^I to cells receiving BTZ treatment further suppressed survival in a dose-dependent manner. Thus, the surviving fraction that was decreased to 67.9 ± 1.3% of controls by 50 nM BTZ alone was further reduced to 47.3 ± 1.2% and 38.4 ± 0.7% of controls (both *P* < 0.001) by adding 50 and 100 μCi of ^131^I, respectively (Fig. 4B).

CD133 and ALDH3A1 expression increased slightly with BTZ treatment alone, to 131.2 ± 8.9% and 141.4 ± 2.5% of controls, respectively (Fig. 4C). However, when ^131^I was added to BTZ treatment, there were markedly reductions of CD133 protein to 26.7 ± 4.0%; ALDH3A1 protein to 36.3 ± 7.4%; and SOX2 protein to 20.2 ± 2.8% of controls (Fig. 4C).

### CT26/NIS-cODC cells enriched for stemness have high radioiodine uptake in vivo

**Figure 5:**
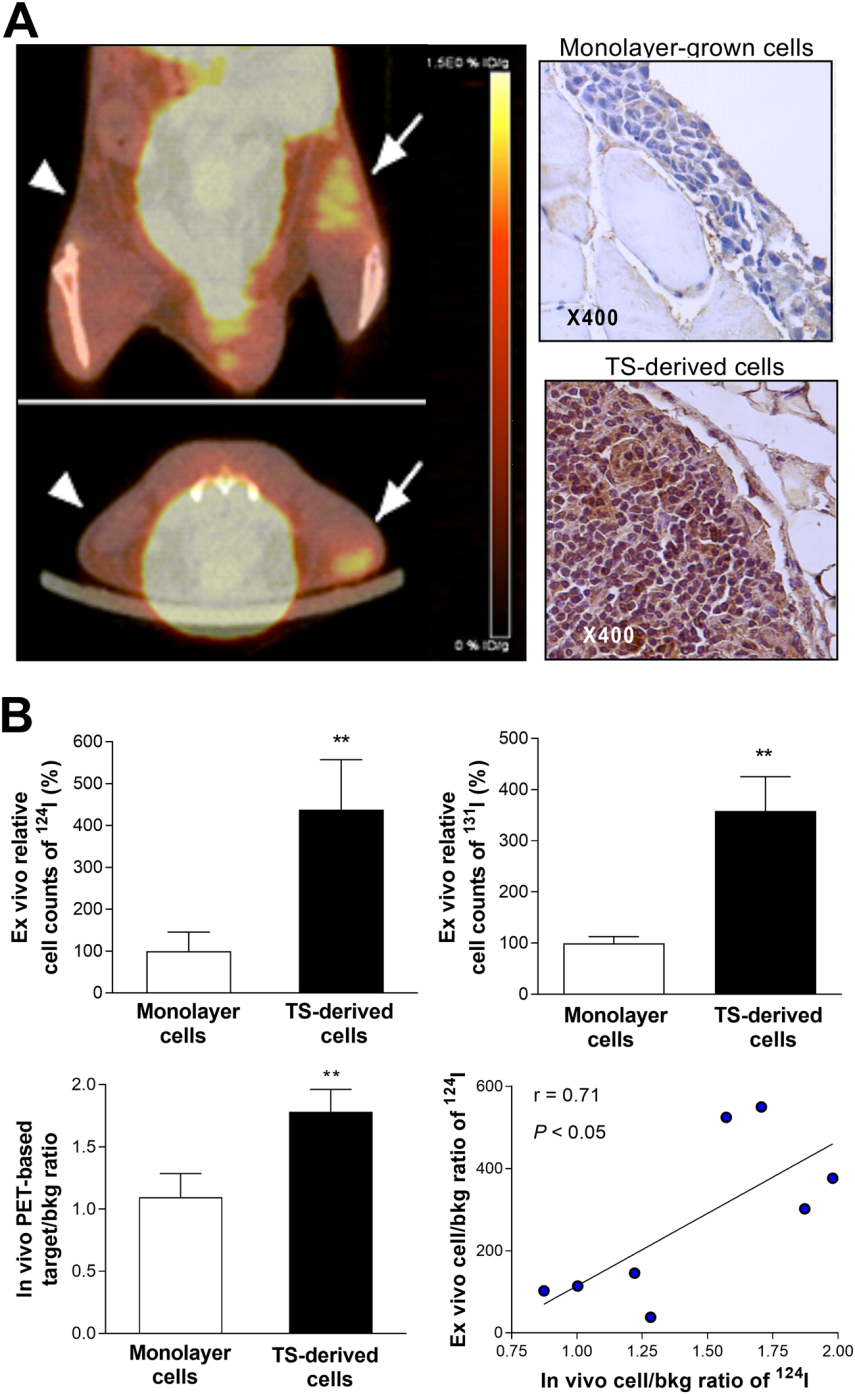
Implanted CT26/NIS-cODC CSCs have increased radioiodine uptake in vivo. (**A**) Coronal and transaxial tomograms of ^124^I PET/CT images from a representative mouse injected with CT26/NIS-cODC cells obtained from monolayers (top; arrowhead) or TS (left; arrow). Immunohistochemistry of NIS expression in thigh tissues injected with cells from monolayers or TS are also shown (right). (**B**) Ex vivo-measured cell-origin relative ^124^I (top, left) and ^131^I counts (top, right). Bars are the mean ± SD of values from mice implanted with cells in either thighs (n = 4 each for ^124^I and ^131^I data). PET image—based target-to-background count ratios (bottom, left). Bars are the mean ± SD of values from four mice implanted with cells in either thighs. The linear correlation between the in vivo and ex vivo measurements is also shown (bottom, right). **P < 0.01 compared to monolayer grown cells. PET/CT: positron emission tomography/computed tomography; TS: tumorsphere.

Prior to tumor model experiments, we confirmed that tumorsphere-derived CSCs have increased radioiodine uptake in living bodies. This was accomplished by implanting the cells into thighs of normal mice followed by intravenous ^124^I injection and positron emission tomography/computed tomography (PET/CT) imaging. The resulting images displayed focal increased radioiodine uptake in regions containing tumorsphere-derived CT26/NIS-cODC CSCs but not in regions with monolayer-grown CT26/NIS-cODC cells (Fig. 5A). Immunohistochemistry confirmed abundance of cancer cells staining strongly positive for NIS protein in the former but not in the latter tissues (Fig. 5A). Region-of-interest analysis confirmed greater target-to-background count ratios for regions containing CSCs compared with those containing monolayer-grown cells (1.78 ± 0.17 vs. 1.09 ± 0.19; *P* < 0.01; Fig. 5B). Ex vivo measured radioactivity counts accredited to the implanted cells showed similar results (438.1 ± 118.7% vs. 100.0 ± 45.0%; *P* < 0.01), and linear regression analysis verified that the PET-based in vivo measurements reliably assessed the level of radioiodine uptake by the implanted cells (Fig. 5B).

Repeat experiments in a separate group of mice using ^131^I instead of ^124^I demonstrated that ex vivo ^131^I counts accredited to the implanted cells was significantly higher at 357.9 ± 134.8% for regions containing tumorsphere-derived CT26/NIS-cODC CSCs compared to regions containing monolayer-grown CT26/NIS-cODC cells (*P* < 0.01). This result is highly consistent with that obtained using ^124^I.

### ^131^I therapy potentiates the efficacy of BTZ treatment against CT26/NIS-cODC tumors

**Figure 6:**
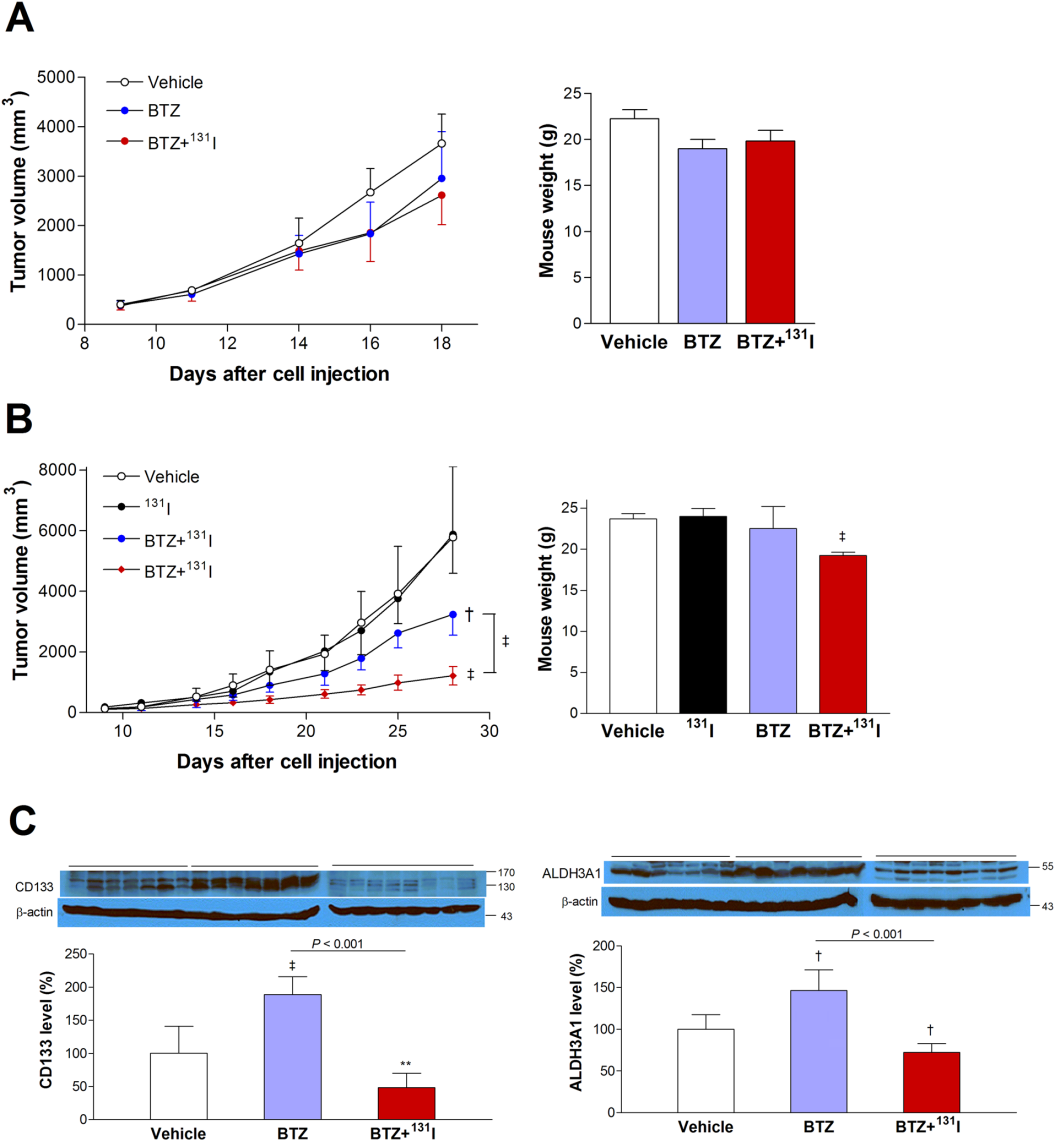
^131^I potentiates BTZ treatment efficacy and suppresses stemness in tumor-bearing mice. (**A**) Growth of CT26 tumors in mice treated with BTZ alone (n = 4) or BTZ plus ^131^I (n = 4) compared to vehicle-injected animals (n = 3). (**B**) Growth of CT26/NIS-cODC tumors in mice treated with ^131^I alone (n = 8), BTZ alone (n = 7) or BTZ plus ^131^I (n = 8), compared to vehicle-injected animals (n = 7). The weights of mice after tumor removal are also shown. All data are the mean ± SD of tumor volume. ^†^*P* < 0.005, ^‡^*P* < 0.001, compared to vehicle-treated controls. (**C**) Western blots for CD133 (left) and ALDH3A1 expression (right) in residual CT26/NIS-cODC tumors after treatment with vehicle (n = 7), BTZ (n = 7), or BTZ plus ^131^I (n = 8). Blots are cropped with single blot parts separated by space. For full length blot pictures, see supplement Fig. 5. Bars are the mean ± SD of % band intensities normalized to β-actin bands. **P < 0.01, ^†^*P* < 0.005, ^‡^*P* < 0.001, compared to vehicle-treated controls. BTZ: bortezomib; ALDH3A1: Aldehyde dehydrogenase 3A1.

When mice were treated with BTZ alone, non-transformed CT26 tumors responded with a slight retardation of growth with a tumor volume of 2,953.4 ± 944.0 mm^3^ by day 18 compared to 3,661.2 ± 590.0 mm^3^ for controls (Fig. 6A). In these animals, adding a single intravenous dose of ^131^I at 7 days of BTZ treatment did not further reduce tumor size (2,613.9 ± 517.8 mm^3^; Fig. 6A).

In mice bearing CT26/NIS-cODC tumors, treatment with ^131^I alone for 28 days did not reduce tumor size compared to that of control mice. Treatment with BTZ alone for 28 days decreased the tumor to 3,235.0 ± 678.2 mm^3^, compared with 6,234.7 ± 2,113.5 mm^3^ for the controls (*P* < 0.005; Fig. 6B). In these animals, a single intravenous dose of ^131^I in addition to BTZ further suppressed tumor volume to 1,212.8 ± 848.2 mm^3^ on day 28 (*P* < 0.001 compared to the BTZ alone; Fig. 6B). Body weight at the end of treatment did not differ between the CT26 tumor-bearing groups and was slightly lower in the BTZ plus ^131^I group for CT26/NIS-cODC tumor-bearing mice (Fig. 6A,B).

### Cancer stemness in vivo is increased by BTZ alone but is suppressed by combining ^131^I therapy

Evaluation of cancer stemness properties in residual tumor tissue after treatment with BTZ alone revealed increased CD133 expression to 188.7 ± 26.7% (*P* < 0.001) and ALDH3A1 expression to 146.5 ± 24.7% of controls (*P* < 0.005; Fig. 6C). However, a single intravenous dose of ^131^I in addition to BTZ significantly decreased these stemness markers. Hence, CD133 expression was suppressed from 188.7 ± 26.7% to 48.4 ± 21.4% of controls (*P* < 0.001) and ALDH3A1 expression was suppressed from 146.5 ± 24.7% to 72.2 ± 10.6% of controls (*P* < 0.001; Fig. 6C).

## Discussion

Treatment resistance remains a serious limitation to our ability to conquer colon cancer^27,28^. Colon tumors contain individual cancer cells that display variable tolerance to chemotherapeutic agents. Hence, initially minor clones that have greater tolerance to the drugs become dominant and promote tumor regrowth^29^. The functional heterogeneity of colon cancers is principally determined by a distinct population of cells with stemness properties^30,31^. Thus, any strategy to cure colon cancer will require a method for specific targeting and the elimination of drug-resistant CSCs.

CSCs differ from the bulk of differentiated cancer cells that have high proteasome activity by having inherently poor proteasome function. This is demonstrated in living cells by specialized protein sequences that are recognized by the proteasomal system for elimination. An example is the well-established cODC degron that undergoes rapid ubiquitin-independent proteasomal degradation. Therefore, cODC sequence-fused proteins are promptly destroyed in cells with normal proteasomal activity but are stable and accumulate in cells with poor proteasome function. In a pioneering work, Vlashi and coworkers constructed and stably expressed a ZsGreen-cODC fusion gene in glioma and breast cancer cells. Increased fluorescent signals allowed the in vitro identification and in vivo tracking of CSCs^23^. The construct also identified CSCs originating from the pancreas, prostate, brain, head and neck, cervix, and lung^20,22,32–34^. Furthermore, the construct showed that breast cancer cells with low proteasome activity produced larger tumors and had more metastatic spread in mice^35^.

In this study, we linked the cODC sequence to the NIS gene to produce a fusion protein that accumulates under poor proteasome function and promotes the intracellular transport of radioiodine for targeted CSC therapy. Blocking proteasome activity of CT26 colon cancer cells stably expressing the NIS-cODC construct resulted in substantial accumulation of NIS protein that was undetectable at baseline because they underwent proteasomal degradation. Our group previously showed that this property of the NIS-cODC system can be exploited for imaging of CT26 tumors under proteasomal inhibition with ^124^I PET^26^. In the present study, CT26 CSCs were found to have intrinsically low proteasome activity even at baseline. Several malignancies harbor a small subset of CSCs with low proteasomal activity^23^, and possible mechanistic links have been identified^36^. CT26/ZsGreen-cODC cells in tumorspheres showed high fluorescence and CT26/NIS-cODC CSCs from tumorspheres showed high NIS protein accumulation. The latter cells had high radioiodine uptake at baseline that was even greater than that of monolayer grown cells under proteasomal suppression. These results confirm that CT26/NIS-cODC CSCs have high radioiodine-avidity due to inherently poor proteasome function.

When CT26/NIS-cODC cells were treated with BTZ for a week, most of the cells were eradicated but there was a small population that remained viable. This is consistent with the notion that cancer cells with low proteasome activity have a propensity to survive treatment and subsequently drive tumor regrowth. For instance, ZsGreen-cODC expression from low proteasome activity has been associated with treatment resistance in malignant cells of the prostate^33^, cervix^34^, bone^37^, and head and neck^30^. Recently, colorectal cancer cells displaying low proteasome activity were associated with stem-like properties and radio-chemoresistance^38^. The link between poor proteasome activity and treatment resistance extends to responses to BTZ, which was previously demonstrated in head and neck^39^ and papillary thyroid CSCs^40^. Also, a new-generation proteasome inhibitor called NPI-0052 failed to eradicate the CSC population in glioblastoma xenografts^41^. Furthermore, lymphoma cells with stemness properties resisted BTZ treatment, whereas the drug suppressed clonogenic activity after the cells had differentiated^42^. Together, these findings indicate that CSCs contribute to BTZ resistance, presumably because they are less dependent on protein degradation for survival than differentiated cancer cells.

Cancer stemness require stabilization of proteins that regulate the pluripotency network^19,43^. Slow protein turnover by proteasomal downregulation prevents the degradation of proteins required for stemness. In this study, CT26 cells that survived BTZ treatment displayed increased expression of CD133, a surface marker highly expressed on CSCs from various origins including colon cancer^44^. BTZ-resistant CT26 cells also showed increased activity of ALDH, an intracellular detoxifying enzyme highly expressed in stem cells and CSCs^45^. P-glycoprotein activity was only slightly increased in these cells, consistent with the notion that BTZ is a poor substrate for p-glycoprotein and that this transporter does not play a large role in BTZ resistance. Importantly, these cells displayed substantially greater tumorsphere-forming capacity. Together, these findings reveal that cancer cells that survive BTZ treatment are enriched with CSCs that have intrinsically poor proteasome function and are insensitive to suppressed protein turnover. In addition to simply selecting for CSCs, proteasome inhibition might even contribute to the acquisition of this phenotype^43^. This points to the importance of novel strategies to eliminate resistant CSCs for the success of proteasome inhibitor therapy.

Perturbed proteasome function that contributes to proteasome inhibitor resistance also offers a unique and attractive opportunity to specifically eliminate treatment-resistant CSCs. As an example, CSCs that expressed the cODC degron fused to the thymidine kinase gene could be targeted by treatment with ganciclovir, which abrogated sphere formation in vitro and caused regression of xenotransplants in vivo^24^. NIS, a selective transmembrane carrier for iodide, not only allows imaging via γ-emitting radioiodine^46^ but also allows the elimination of target cells via β-emitting ^131^I^47^. NIS provides a versatile system for targeting cells in living bodies because it does not deter basic cell physiology or elicit an immune response in vivo. It is not a foreign protein but is normally expressed in only a few tissues. Furthermore, various radioiodine substrates for NIS-mediated uptake are already widely applied for clinical use and are readily available without any radiochemical synthesis^46,47^.

In our in vitro experiments, treating CT26/NIS-cODC cells with ^131^I plus BTZ suppressed cell survival to a significantly greater degree than BTZ alone. Furthermore, the stemness markers ALDH3A1 and CD133 were increased by treatment with BTZ alone but were substantially decreased when ^131^I therapy was added. The stemness marker SOX2 was also substantially reduced by ^131^I plus BTZ treatment. These results indicate that ^131^I therapy eliminates CSCs that survive BTZ treatment but accumulate NIS protein because of low proteasome activity.

Before performing ^131^I therapy in tumor-bearing mice, we first confirmed that CT26/NIS-cODC CSCs are radioiodine avid in living bodies by comparing ^124^I uptake after implantation in mice. Because CSCs become greatly outnumbered by differentiated cancer cells over time, imaging was performed on the day of implantation for this experiment. PET imaging confirmed high ^124^I uptake in CT26/NIS-cODC CSCs but not ordinary CT26/NIS-cODC cells. The accuracy of PET-based assessment of uptake was confirmed by correlation with ex vivo measured uptake. This confirmed that CT26/NIS-cODC CSCs have stabilized NIS accumulation that instigates high radioiodine uptake in living bodies.

Finally, we investigated the therapeutic capacity of the NIS-cODC system in tumor-bearing mice. Treatment with BTZ alone produced only modest growth retardation in both CT26 and CT26/NIS-cODC tumors. Addition of ^131^I therapy during BTZ treatment further suppressed the growth of CT26/NIS-cODC tumors but not CT26 tumors. Furthermore, tumor expression of stemness markers, which increased after treatment with BTZ alone, decreased significantly when ^131^I therapy was added.

In conclusion, this study demonstrates that radioiodine targeted elimination of CSCs is an efficient strategy to overcome tumor resistance to BTZ therapy. This can be accomplished by stabilization and accumulation NIS-cODC fusion protein via poor proteasome activity, which is a biologic characteristic of CSCs.

## Materials and methods

### Cell culture and preparation of stably expressing cell lines

CT26 mouse colon cancer cells (ATCC, #CRL-2638, USA) were maintained in RPMI (Lonza, Basel, Switzerland) supplemented with 10% fetal bovine serum (FBS; Serana, Germany) and 1% penicillin/streptomycin (Gibco Laboratories, USA) at 37 °C and 5% CO_2_ in a humidified atmosphere. CT26 cells that constitutively expressed NIS-cODC or ZsGreen-cODC fusion protein were prepared as previously described^26^). Briefly, the cells were infected with pQCXIN retroviral particles containing the NIS-cODC or ZsGreen-cODC sequences, and single-cell clones with stable expression (CT26/NIS-cODC or CT26/ZsGreen-cODC cells) were selected with geneticin (Gibco Laboratories). Because the cODC sequence is a target for ubiquitin-independent degradation by the 26S proteasome, the accumulation of NIS-cODC instigates radioiodine uptake and identifies cells with poor proteasome function. Similarly, accumulation of ZsGreen-cODC identifies cells with poor proteasome activity by emitting green fluorescence. All cell lines were routinely tested for mycoplasma.

### Proteasome activity assay

Cells in 6-well plates (1.2 × 10^6^) were washed and collected by scraping with 0.5% NP-40 in distilled water. After incubation for 30 min at − 20 °C and centrifugation at 13,000 rpm for 10 min at 4 °C, the proteasome activity in the supernatants was measured with a fluorometric assay kit (Bio Vision, CA). Briefly, cell extracts were mixed with 100 μl of assay buffer and 1 μl of Suc-LLVY-7-amino-4-methylcoumarin (Suc-LLVY-AMC) substrate. Fluorescence was measured at 350 nm excitation and 440 nm emission wavelengths in a microplate reader at 37 °C for 30 min. Proteasome activity was measured as the rate increase in fluorescent signals caused by Suc-LLVY-AMC degradation^26^.

### CSC enrichment through tumorsphere formation and limiting dilution assays

Cells were enriched for cancer stemness using tumorsphere formation. Briefly, cells were grown on an ultra-low attachment culture plate (Corning, NY) in CSC selection medium. The selection medium was prepared in a DMEM/F12 base that contained 0.4% bovine serum albumin, B27 supplement (Gibco Laboratories), 5 μg/ml bovine insulin, 4 μg/ml heparin, 20 ng/ml fibroblast growth factor 2, and 20 ng/ml epidermal growth factor. Tumorspheres were efficiently formed under this condition and were grown for 10 days, with fresh medium provided every 2 to 3 days.

Tumorsphere-forming capacity was evaluated by limiting dilution assays. After dilution, 1, 10, 100, and 1,000 cells were seeded and cultured under the sphere formation conditions listed above. After 10 days, the number of tumorspheres was counted under a microscope.

### Cellular radioiodine uptake measurement

Monolayer cells were incubated for 1 h with 74 kBq of ^125^I (Perkin Elmer, MA) added to the culture medium in 5% CO_2_ at 37 °C. Cells were rapidly washed twice with cold PBS, lysed with 0.1N NaOH, and measured for cell-bound radioactivity on a γ-counter (Wallac). Uptake levels were normalized to the protein content of each sample^26^. For tumorsphere radioiodine uptake measurement, tumorspheres were collected and digested with trypsin-EDTA for 10 min at 37 °C. Single cells were washed and seeded on a Matrigel-coated 24-well culture plate with RPMI medium containing 20% FBS. Control cells did not undergo tumorsphere formation but were seeded under identical conditions. 48 h after the cell seeding, the radioiodine uptake experiment was carried out as described above.

### In vitro therapy and sulforhodamine B (SRB) survival assay

Cells on a 96-well plate were treated with BTZ (Calbiochem, MA) by adding the drug to the culture medium. For radioiodine therapy, 50 μCi or 100 μCi of ^131^I was added to the medium in each well, and the medium was replaced with fresh medium after 9 h of incubation at 37 °C.

The surviving cell content was measured using SRB assays^48^. Briefly, treated and untreated cells were fixed with 10% (wt/vol) trichloroacetic acid at 4 °C and stained with SRB dye (Sigma-Aldrich) for 30 min. Excess dye was removed by repeated washing with 1% (v/v) acetic acid. Protein-bound dye was eventually dissolved in 10 mM Tris base solution and subjected to spectrophotometric measurement of absorbance at 510 nm on a microplate reader.

### Aldehyde dehydrogenase (ALDH) activity assay

ALDH activity was measured with an Aldefluor assay kit (Stemcell Tech, BC, Canada) according to the manufacturer’s manual^49^. Cells were resuspended in Aldefluor assay buffer containing 1 μM Aldefluor dye. ALDH activity was blocked with 15 μM of the selective inhibitor DEAB. After incubation at 37 °C for 30 min, cells were washed with assay buffer, and 10,000 cells were analyzed on a FACS Calibur flow cytometer with CellQuest software (Becton-Dickinson, NJ).

### P-glycoprotein activity assay

The activity of p-glycoprotein-mediated drug efflux was assessed by Efluxx-ID Green assays (ENZO Life Sciences, Lȍrrach, Germany) following the manufacturer’s protocol^50^. Briefly, cells harvested by trypsinization were pre-incubated for 5 min at room temperature in PBS containing 2% FBS. The buffer contained 20 μM verapamil, an MDR1 inhibitor, in DMSO or DMSO (vehicle). After Efluxx-ID Green dye was added, the cells were incubated for 30 min at 37 °C. Then, 10,000 cells per sample underwent FACS analysis on a Calibur flow-cytometer using CellQuest software (Becton-Dickinson, NJ). Efluxx-ID Green was excited at 490 nm, and fluorescent emissions were detected at 514 nm.

### PET imaging and radioiodine uptake measurements of implanted cancer cells

Before performing murine tumor model experiments, we evaluated whether stem-like cancer cells absorbed increased radioiodine in living bodies. For this experiment, CT26/NIS-cODC cells were prepared by trypsinization of tumorspheres followed by the stabilization of cells in culture medium at 37 °C for 30 min before implantation. Normal BALB/c male 5 weeks aged mice (Orient bio, Korea) who had not received BTZ treatment (n = 4) were injected with cells with and without CSC enrichment on the right and left thigh, respectively. After 30 min, the animals were injected with 13.0 MBq of ^124^I, and PET/CT images were acquired 1 h later. Quantitative analysis of the radioiodine uptake on PET images was performed using the tumor-to-background (shoulder region) ratio as an index of uptake.

Immediately after PET imaging, the animals were sacrificed by cervical dislocation, and thigh tissue injected with tumor cells and shoulder tissue were extracted, weighed, and measured for ^124^I activity. For ex vivo quantification, ^124^I counts attributable to cancer cells were calculated using the following formula. Counts from cancer cells = total thigh tissue counts—background tissue counts. The latter was estimated from shoulder tissue counts corrected for weight (the thigh-to-shoulder muscle weight ratio). The resulting count from cancer cells per weight of target tissue was finally divided by the count per weight of shoulder tissue to obtain the relative radioactive count ratio as an index of cancer cell uptake.

This experiment was repeated in a separate group of 5 week-old normal male BALB/c mice (n = 4) under the same conditions but using ^131^I instead of ^124^I. Cells were prepared and injected into thigh muscles of the mice and ^131^I of 5.5 MBq was intravenously injected 30 min later. Tissues were then extracted, weighed, and ^131^I attributable to cancer cells were calculated as above.

### Mouse xenograft model and treatment

Tumor models were prepared in BALB/c male 5 weeks aged mice (Orient bio, Korea) by subcutaneous injection of 3.2 × 10^6^ non-transformed CT26 cells or CT26/NIS-cODC cells into the right shoulder region. After 4 days, animals with early tumors of similar sizes were randomly divided into treatment groups: DMSO vehicle group (n = 3 for CT26; n = 7 for CT26/NIS-cODC), ^131^I alone group (n = 8 for CT26/NIS-cODC), BTZ alone group (n = 4 for CT26; n = 7 for CT26/NIS-cODC), and BTZ plus ^131^I treatment group (n = 4 for CT26; n = 8 for CT26/NIS-cODC). BTZ was intraperitoneally injected (2 mg/kg) three times per week for 28 days. ^131^I treatment was given as a single 800 μCi dose by intravenous injection 11 days after cancer cell injection. Mice were assessed for body weight and tumor size at the time of each BTZ treatment. After the end of treatment, the mice were sacrificed by cervical dislocation. Tumor tissues were excised, and the weight of the mice after tumor removal was measured. All animal experiments were in accordance with the National Institutes of Health Guide for Care and Use of Laboratory Animals and approved by the appropriate institutional committee.

### Immunoblotting

Immunoblotting was performed as previously described^26^. Cultured cells and tumor tissues were lysed with cold protein extraction solution (PRO-PREP; Intron, Korea) and T-PER tissue protein extraction solution (Thermo Fisher Scientific), respectively, that contained a protease inhibitor cocktail (Sigma Aldrich). After protein assays, 30 μg of samples were separated by electrophoresis on a 10% sodium dodecyl sulfate polyacrylamide gel, followed by transfer to a polyvinylidene difluoride membrane. The membrane was blocked with 5% nonfat milk in Tris-buffered saline and polysorbate-20 for 1 h at room temperature and incubated overnight at 4 °C with primary antibodies against NIS (Santa Cruz Biotechnology, #sc-134515), β-actin (Santa Cruz Biotechnology, sc-47778), CD133 (abcam, #ab16518), ALDH3A1 (abcam, #ab76976), or SOX2 (abcam, #ab92494). The membranes were then incubated for 1 h at room temperature with a secondary anti-rabbit IgG antibody (Cell Signaling, #7074) for NIS, CD133, ALDH3A1, and SOX2 or a secondary anti-mouse IgG antibody (Cell Signaling, #7076) for β-actin. Immune reactive proteins were detected by chemiluminescence, and band intensities were quantified on a GS-800 densitometer using Quantity One software (Bio-Rad Laboratories, CA).

### Immunohistochemistry staining

Immunohistochemistry staining of formalin-fixed and paraffin-embedded tissues was performed as previously described^26^. After micro-sectioning at 4 μm thickness, the sections underwent heat-induced antigen retrieval for 3 min in EDTA buffer (pH 9.0; Dako, Carpinteria, CA). Sectioned slides were then incubated overnight with anti-human NIS (1:100 dilution; Santa Cruz Biotechnology, #sc-134515) at 4 °C. This was followed by incubation with HRP-labeled polymer-conjugated secondary antibodies against rabbit IgG (Dako) for 30 min at room temperature. A color reaction was induced using a ready-to-use 3,3′-diaminobenzidine substrate-chromogen solution (Dako) for 5 min, followed by washing with distilled water. Finally, sections were lightly counterstained with Mayer’s hematoxylin for 30 s before dehydration and mounting.

### Statistical analysis

Data are presented as the mean ± SD. The significance of differences between groups was analyzed by two-tailed unpaired student’s *t*-tests for two groups and ANOVA with Tukey’s post-hoc tests for three or more groups. *P* values < 0.05 were considered statistically significant.

### Ethical approval

All animal experiments were performed according to the National Institutes of Health Guide for Care and Use of Laboratory Animals and were approved by the Institutional Animal Care and Use Committee of Samsung Medical Center.

## Supplementary information

Supplementary Information.

## Data Availability

All data generated or analyzed during this study are included in this published article.
