# Supplementary material for: Targeting poor proteasomal function with radioiodine eliminates CT26 colon cancer stem cells resistant to bortezomib therapy
Source: Sci Rep. 2020 Aug 31;10:14308. doi: 10.1038/s41598-020-71366-3 (PMC7459321; doi:10.1038/s41598-020-71366-3)
Supplement: Supplementary file 1 — Supplementary Information. [file 41598_2020_71366_MOESM1_ESM.docx]

**Supplementary information**

**Targeting Poor Proteasomal Function with Radioiodine Eliminates CT26 Colon Cancer Stem Cells Resistant to Bortezomib Therapy**

**(Short Running Title: Radioiodine Therapy of Cancer Stem Cells)**

Jin Hee Lee*^1,2^*, Kyung-Ho Jung*^1,2^*, Jin Won Park*^3^*,

Seung Hwan Moon*^1^*, Young Seok Cho*^1^*, Kyung-Han Lee*^1,2^*^,*^

^1^Department of Nuclear Medicine, Samsung Medical Center, Sungkyunkwan University School of Medicine, Seoul, Korea; ^2^Department of Health Sciences and Technology, SAIHST, Sungkyunkwan University, Seoul, Korea, ^3^Scripps Korea Antibody Institute, Chuncheon-si, Gangwon-do, Korea

***Corresponding Author (for reprints):** Kyung-Han Lee, MD, PhD.

Nuclear Medicine, Samsung Medical Center, 50 Ilwon-dong, Gangnam-gu, Seoul, Korea.

Tel: 82-2-3410-2630; Fax: 82-2-3410-2639; [khnm.lee@samsung.com](mailto:khnm.lee@samsung.com)

Supplemental Figure 1: Full length blots from Figure 1B. Western blots of protein from cell lysate for detection of NIS and β-actin.

Supplemental Figure 2: Full length blots from Figure 2A. Western blots of protein from cell lysate for detection of CD133, NIS and β-actin.

Supplemental Figure 3: Full length blot from Figure 3B. Western blot of protein from cell lysate for detection of CD133 and β-actin.

Supplemental Figure 4: Full length blot from Figure 4C. Western blots of protein from cell lysate for detection of CD133, ALDH3A1, SOX2 and β-actin.

Supplemental Figure 5: Full length blot from Figure 6C. Western blots of protein from cell lysate for detection of CD133, ALDH3A1 and β-actin.
